# A Closer Look at the Cellular and Molecular Components of the Deep/Muscular Fasciae

**DOI:** 10.3390/ijms22031411

**Published:** 2021-01-30

**Authors:** Caterina Fede, Carmelo Pirri, Chenglei Fan, Lucia Petrelli, Diego Guidolin, Raffaele De Caro, Carla Stecco

**Affiliations:** Department of Neurosciences, Institute of Human Anatomy, University of Padova, 35121 Padua, Italy; yutianfan1218@163.com (C.F.); lucia.petrelli@unipd.it (L.P.); diego.guidolin@unipd.it (D.G.); rdecaro@unipd.it (R.D.C.); carla.stecco@unipd.it (C.S.)

**Keywords:** fascia, cells, extracellular matrix, nerve

## Abstract

The fascia can be defined as a dynamic highly complex connective tissue network composed of different types of cells embedded in the extracellular matrix and nervous fibers: each component plays a specific role in the fascial system changing and responding to stimuli in different ways. This review intends to discuss the various components of the fascia and their specific roles; this will be carried out in the effort to shed light on the mechanisms by which they affect the entire network and all body systems. A clear understanding of fascial anatomy from a microscopic viewpoint can further elucidate its physiological and pathological characteristics and facilitate the identification of appropriate treatment strategies.

## 1. Introduction

Researchers and clinicians have dedicated quite a bit of attention to fascial tissues over the last few decades; currently, their interest has been focused on their anatomical and pathophysiological features. Ultrasound (US) [1,2,3,4,5] and magnetic resonance imaging (MRI) [6] have taken important steps forward that permit us to study the dynamic structure and the alterations of the tissue. While in vivo studies are critical for analyzing some fascial properties, such as stiffness, thickness, gliding and relationships with other anatomical structures, the fasciae also need to be studied from a microscopic point of view. 

Many morphological descriptions of the fasciae can be found in the literature. According to some authors [7,8], the deep muscular fascia consists of a dense, regular connective tissue similar to aponeurosis, characterized by extremely ordinate, parallel bundles of inelastic collagen fibers. Another definition that can be found in the same edition of Gray’s Anatomy [8] indicates that it is loose irregular connective tissue. According to others [9,10], it is formed by numerous laminae of dense connective tissue in which the collagen fibers can be aligned in more than one direction. For yet others [11], the laminae are difficult to distinguish because the collagen fibers often pass from one lamina to an adjacent one. Additionally, according to some investigators [12], the deep/muscular fasciae of the lower limbs are composed of intertwined bundles of collagen fibers.

Some investigators [13,14,15] have distinguished between the various types of fasciae (superficial, deep/muscular and visceral fasciae), while others have focused on their continuity, pointing out that their densities but not basic structures are variable. According to Guimberteau et al. [16], fascia is a structure that evolves hierarchically from a one cell embryo to the organism, and it is constantly adapting to new stresses to meet the organism’s structural demands. A similar description was given by Levin and Martin [17] who considered a fascia to be a tension network, with all the collagen inherently stressed—the so-called “pre-stress” of biologic tissues. 

The current study intends to review the information gathered until now about the microscopic anatomy and the cellular components of the fasciae as well as the composition of the extracellular matrix and the receptor expression in fascial tissue. It will focus on characterizing the components of the fasciae in an effort to examine the factors which regulate their morphological features and how they adapt to different types of stress, conditions, pathologies and other variables. 

## 2. Classification of the Fasciae

At the Fourth International Fascia Research congress, which was held near Washington, DC in September of 2015, two definitions of fascia were proposed: it was, in fact, called both “a fascia” and “the fascial system” [18]. A fascia is “a sheath, a sheet, or any other dissectible aggregations of connective tissue that forms beneath the skin to attach, enclose, and separate muscles and other internal organs”. The fascial system “consists of the three-dimensional continuum of soft, collagen-containing, loose and dense fibrous connective tissues that permeate the body, providing an environment that enables all body systems to operate in an integrated manner”. The first is clearly a morphological/anatomical definition, while the second is a functional one. As the current study intends to examine the fascia primarily from an anatomical viewpoint, we will dedicate ourselves to exploring the first definition. 

The fasciae can be distinguished into three main types depending on their histological characteristics and anatomical relationships:

- Superficial fascia, which is found directly under the skin and superficial adipose layer. It can show stratification both grossly and microscopically. It is conventionally described as being made up of membranous layers with loosely packed interwoven collagen and elastic fibers more superficial than other types and containing more elastic fibers [19];
- Deep/muscular fasciae: depending on their orientations, composition architectures, and anatomical locations, the two main types of deep/muscular fasciae are the aponeurotic fasciae and the epimysial fasciae [13,20]. The former refers to all the “well-defined fibrous sheaths that cover and keep in place a group of muscles or serve for the insertion of a broad muscle”, as in the case of the deep fasciae of the limbs, the thoracolumbar fascia and the rectus sheath of the abdomen [21]. The latter refers to the connective tissue sheath surrounding skeletal muscle and, in some cases, directly connected to the periosteum of the bones as in the case of the deep fascia of the trunk and the epimysium of the limb muscles;
- Visceral fasciae, which are all the fasciae closely connected to individual organs and giving shape to them, support the parenchyma as well as all the fibrous sheets forming the compartments for the organs and connect them to the musculoskeletal system [22];
- Neural fasciae, which are all the meningeal layers and the connective tissues that envelop the peripheral nerves.

The deep/muscular fasciae have been receiving a great deal of attention over recent years in view of their possible role in proprioception, motor coordination and pain. Given the relevance of these three elements, the current study will focus on the microscopic characteristics of the muscular fasciae. 

## 3. Thickness of the Deep/Muscular Fasciae

Morphometrically, the aponeurotic fasciae of the limbs present a mean thickness of 1 mm (590–1453 μm). The fasciae are thicker in the inferior limbs and posteriorly. The deep fascia of the thigh is thinner in the proximal region and thicker near the knee (mean thickness = 926 μm, SD ± 156 μm). In the lateral region, it is reinforced by the iliotibial tract. In the superior limbs, the brachial fascia presents a mean thickness of 863 μm (SD ± 77 μm); it is thinner in the anterior region and thicker in the posterior one. The antebrachial fascia has a mean thickness of 755 μm (SD ± 115 μm) and presents two reinforcements: the lacertus fibrosus and the retinacula of the wrist. These dimensions are consistent with those reported by Pirri et al. [23], whose in vivo data were obtained via ultrasound evaluation. This study, which evaluated the deep fasciae of different regions of the thigh, uncovered a mean thickness of 556.8 ± 176.2 µm in the anterior compartment, of 820.4 ± 201 µm in the medial one, of 1112 ± 237.9 µm in the lateral one, and of 730.4 ± 186.5 µm in the posterior one. When Wilke et al. [24] performed an ultrasound evaluation of the various fasciae in younger and older age groups, they found that young adults exhibited higher fascia thickness of the anterior and posterior lower leg, anterior thigh and abdominal wall (+12.3–25.8%, *p* < 0.05). The older groups instead showed higher thickness in the thoracolumbar fascia (2.35 versus 1.33 mm, +40.0–76.7%, *p* < 0.05). Correlations have been found between body mass and fascia thickness (τ = 0.45–0.75, *p* < 0.05) as well as between flexibility and fascia thickness (τ = 0.38–0.42, *p* < 0.05). We reported in one of our previous works that the thickness of the rectus sheath was significantly thicker in women who had undergone a caesarean section with respect to those who had undergone a vaginal delivery and/or healthy nulliparous women [25]. 

Reporting on the pectoral fascia of the epimysial fasciae, Stecco et al. [26] described a mean thickness of 150–200 µm. These investigators also noted that the fasciae were increasingly thicker towards the cranio-caudal direction. In fact, there was a mean thickness of 131 mm in the subclavicular region, 182 mm in the mammary region and 578 mm in the inferior thorax one. Wilke et al.’s [24] in vivo study evaluating the epimysial fascia of the erector spinae muscles found a mean thickness of 0.62 mm (0.4–1.75 mm). 

Ultimately, the thickness of the muscular fascia seems to vary depending on its anatomical location, the individual’s body mass index (BMI), age and physiopathologic condition.

## 4. Microscopic Anatomy of the Deep/Muscular Fasciae 

**Figure 1:**
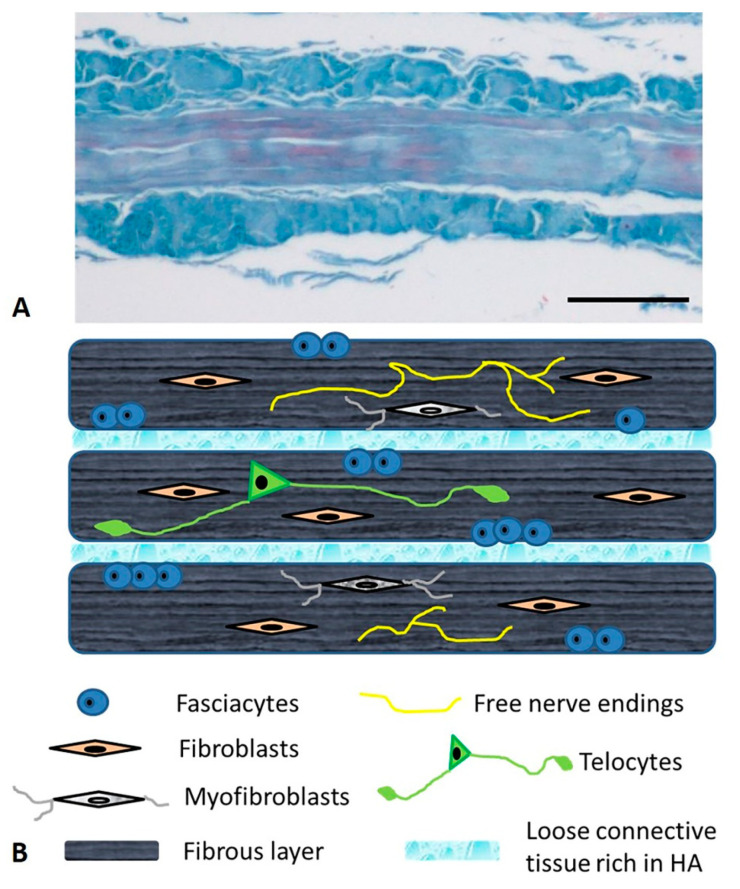
Microscopic anatomy of the human deep/muscular fascia. (**A**): Human fascia lata of the thigh—Azan Mallory staining Bar 250 µm; (**B**): schematic representation.

Many fibrous bundles running in different directions are macroscopically visible in the aponeurotic fasciae. This is why the aponeurotic fasciae were initially classified as irregular dense connective tissues. However, recent works [26,27,28] have demonstrated that the aponeurotic fasciae consist of two or three layers of parallel collagen fiber bundles, with each layer having a mean thickness of 277 µm (±SD 86.1 µm). The collagen fibers of adjacent layers may have different orientations. Each layer is separated from the adjacent one by a thin layer of loose connective tissue that permits them to slide over one another. Two thin layers (mean thickness 23 μm) of fibroelastic tissue are present on the external and internal sides of all the fasciae [26] (Figure 1).

The epimysial fasciae are formed by undulated collagen fibers, arranged more or less transversely with respect to the underlying muscle, and they present an elevated number of elastic fibers which form an irregular mesh. The fascial layers providing intermuscular connections are able to transmit muscular force between adjacent synergistic muscles. The estimated percentage of elastic, with respect to collagen fibers, is approximately 15% [26]. Purslow et al. demonstrated that the epimysium has the same organization as the aponeurotic fasciae which consist of two or three sublayers of connective tissue and they described three sublayers of the epimysium: the internal, the middle and the external sublayers [29,30].

From a microanatomical point of view, the deep fascia is composed of various kinds of cells embedded in the extracellular matrix and there is an abundance of nervous fibers [31]. While the nervous components define the sensitive role of the fascial tissue, the cellular ones adapt the fascia to varying conditions, defining its metabolic properties and synthesizing the extracellular matrix. The latter is made up of two components: protein fibers and a water component. The former is essentially collagen type I, with some fibers of collagen type III and elastic fibers, which give support and organization to the structure. The latter, which is the ground substance, is a water-rich gelatinous substance containing abundant glycosaminoglycans, which confers turgor and flexibility to the tissue, permits the gliding property and transports metabolic material (Figure 1). 

## 5. The Cells of the Fascial Tissue

The predominant cell population of the fascial tissue are *fibroblasts* [32,33]; their principal role is to maintain the structural integrity and organization of the tissue as they are involved in mechanotransduction and in secreting precursors of the extracellular matrix. As opposed to epithelial tissue, they are distributed randomly and not organized in flat monolayers or restricted to one side of the tissue. 

**Figure 2:**
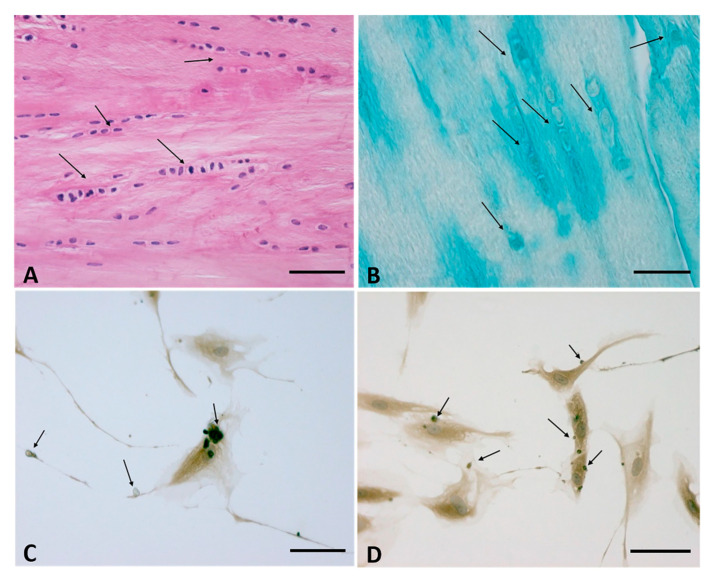
(**A**,**B**): Fasciacytes in Human fascia. (**A**): Hematoxylin and Eosin staining, Bar 50 μm—the nuclei were counterstained with hematoxylin; the Fasciacytes are highlighted by black arrows. (**B**): Alcian Blue/MgCl_2_ 0.05 M, Bar 25 μm, the Fasciacytes are highlighted by black arrows. The fasciacytes have round nuclei, cytoplasm restricted to the perinuclear region, and smaller and less elongated cellular processes. (**C**,**D**): Human Monolayer Fascial fibroblasts treated with HU-308: Immunostaining with HABP Antibody. Arrows indicate HA positive vesicles. Bar 50 µm.

The *fasciacytes*, small clusters of rounded cells along the surface of each fascial layer in the fascia lata, are specialized to the synthesis of hyaluronan (HA) of the ground substance [34]. They are fibroblast-like cells that are positive for the fibroblast marker vimentin, and their negativity to anti-CD68 has proven that they are not derived from the monocyte/macrophage lineage [35], as is the case for other HA-secreting cells, such as synoviocyte type B cells [36] or the hyalocytes of the eye [37,38]. The S100-A4-protein positive cells are markers that permit us to differentiate the cells from the classical fascial fibroblasts and to make the connection with the chondrocytes. They constitute markers for chondroid metaplasia or rather the reversible transformation towards a chondroid-like cell, despite the negative reaction to the marker of the chondrocyte family, collagen II [39]. In conclusion, it is a new type of cell, with a specialized function of HA synthesis and a rounded morphology; its cytoplasm is restricted to the perinuclear region, and it has smaller, less elongated cellular processes (Figure 2).

In normal healthy fascia, about 30% of the fibroblasts are fasciacytes, although this percentage may vary, depending on the stimuli to which the fascial tissue is subjected. The number and the specific localization of these cells in the sublayers of the fascial tissue is strictly correlated to their function: the fibroblasts produce the fibrous component (collagen and elastic fibers) and play a role in regulating force transmission at a distance. The fasciacytes instead produce hyaluronan, which allows fascial gliding between adjacent fascial sublayers. 

The fasciae also contain *myofibroblast* cells which are specialized fibroblasts exhibiting contractile activities that regulate the basal tone of the tissue. The cell density of myofibroblasts in human fascia is different between body sites [40]. “In the human lumbar fascia [median 1.52% (IQR 0.17–4.89%), *n* = 12], it was found to be considerably higher than in the human plantar fascia and the fascia lata [0% (0–0%, *n* = 11, *p* = 0.003) versus 0% (0–0.03%, *n* = 12, *p* = 0.003)]” [40].The observed density of MFBs in human lumbar fascia in this study could possibly be associated with an augmented occurrence of (micro)injuries and related cellular repair processes in human lumbar fasciae. The contractile activity takes place via the adherens junctions, which open mechanosensitive ion channels in adjacent cells, resulting in Ca^2+^ influx [41]. The latter induces a contraction that can feed back on the first myofibroblast and/or stimulate other contacting myofibroblasts [41]. This coordination would gain importance during tissue remodeling when a high number of myofibroblasts contract the extracellular matrix (ECM) at the same time. The myofibroblasts are known to play a role in some pathological fibrotic contractures that affect the fascia. They may present abnormal proliferation, leading to a pathological contracture called Dupuytren disease that affects the palmar and digital fascia of the hand [42]. Some studies have recently outlined their role in normal fascial tissues; when viewed during a time-window of several minutes and longer, cellular contractions can impact motoneuronal coordination, musculoskeletal dynamics and regulation of fascial stiffness, inducing substantial contractures (~1 cm per month) [40,43]. 

**Figure 3:**
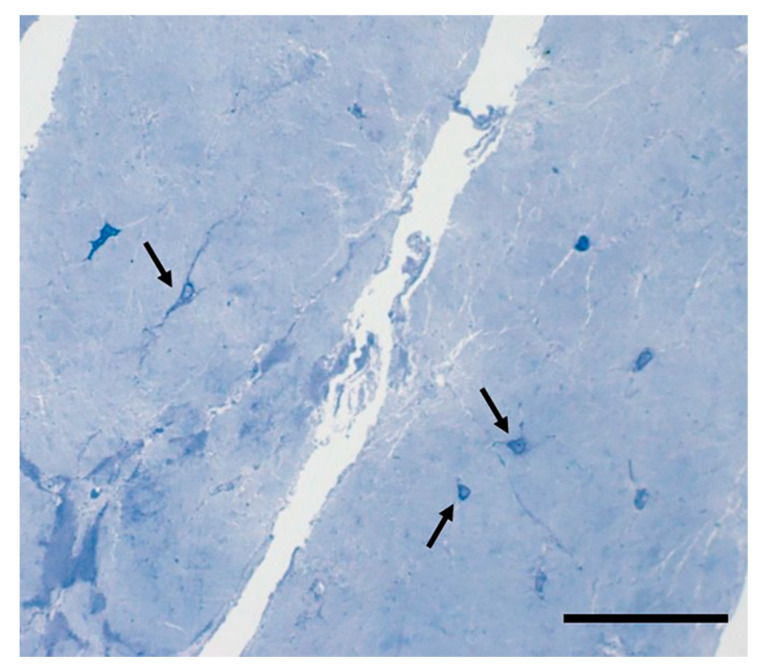
Telocyte in Human fascia-Semithin sections, Toluidine blue staining. Bar 25 µm. The telocytes are highlighted by black arrows.

Recent studies have also brought to light fascial structures containing *telocytes*, specialized connective tissue cells possessing long thin extensions called telopodes (Figure 3). Dawidowicz et al., who first described the presence of telocytes in the fascia lata [44,45], called them a “network in network” system, in view of the complex three-dimensional communication system that they form in the interstitial extracellular matrix. Telocytes have recently been identified in the tensor fascia lata, the crural fascia of the leg, the plantar fascia and the thoracolumbar fascia [46]. Although their specific role and distribution is still under investigation, these cells are believed to be involved in cell repair, regeneration, remodeling, immune control and cell communication via cell junctions or extracellular vesicles [47]; as such they probably play an important role in regulating myofascial pain and fascial disorders. 

## 6. The Nervous Fibers of the Fascia

**Figure 4:**
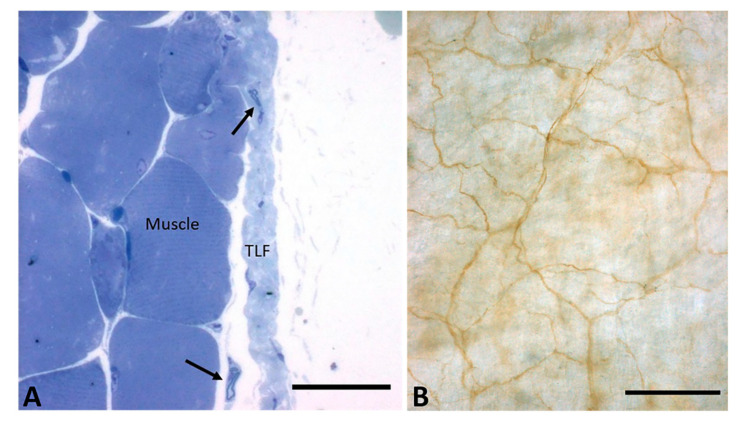
(**A**): Nerve fibers in a mouse’s thoraco-lumbar fascia, semithin sections, Toluidine blue staining (Bar 25 µm); (**B**): network innervation (brown staining lines, S100 Antibody) in thoracolumbar fascia layers. Bar 200 µm. TLF: thoracolumbar fascia. Black arrows: nerve fibers.

One of our recent works [15] demonstrated that the fasciae contain a large thin network of free nerve endings that may play a role in pain perception and regulation (Figure 4). The superficial fascia of the human hip was found to be the second most highly innervated tissue after the skin, with a density of 33 ± 2.5/cm^2^ and a mean nerve size of 19.1 ± 7.2 μm. The tendon was the least innervated. Different types of sensory receptors are present in the fasciae: muscle spindles (epimysial fasciae), the Golgi tendon organ, Pacini and Ruffini corpuscles (aponeurotic fasciae), and an extensive network of free nerve endings. Each has a specific function depending on its structure and activating stimuli [13]. While Pacinian corpuscles are rapidly adapting mechanoreceptors sensitive to changes in stimulation, Ruffini corpuscles are slowly adapting mechanoreceptors that are highly sensitive to shear loading. The innervation of the fascial tissue is not homogeneous: while the visceral fascia is rich in autonomic innervation [22], the superficial fasciae share a link with skin mechanoreceptors and thermoreceptors, and the deep fascia plays a role in proprioception. Ruffini and Pacini corpuscles are principally located in the superficial fascia where it is attached to the deep fascia, as in the palmar [48], the plantar fasciae [49], the wrist [50] and the ankle retinacula [51]. The nerve elements within the superficial fascia in these areas probably reinforce the proprioceptive stimuli of the deep fascia. No Pacini and Ruffini corpuscles have been found in the thoracolumbar fascia [52] or fascia lata [15]. Innervations within the same fascia vary depending on the fibrous sublayers. Tesarz et al. [28], for example, demonstrated that the outer sublayer of the posterior layer of the thoracolumbar fascia is the most innerved portion. The middle one, corresponding to the tendon of the latissimus dorsi muscle, is instead largely free of endings. 

The role of fascial tissue in connection to pain has become an increasingly important issue. Taguchi and coauthors [53] uncovered the presence of peptidergic and nonpeptidergic axons of unmyelinated C-fibers with nerve terminals in rat crural fasciae. Schilder et al. [54] clearly demonstrated that even more than the muscles and the subcutis, the deep fascia plays a significant role in the generation of pain symptoms. Injections of hypertonic saline into the deep fascia resulted in longer pain duration and higher peak pain ratings than injections into the subcutaneous tissue or muscle. Mense and Hoheisel [55] demonstrated that the densities of CGRP- and SP-positive fibers were significantly higher in the inner and outer layers of the inflamed fascia. No inflammation-induced change occurred in the thick middle layer. 

The fasciae also seem to be connected to autonomic innervation. According to Mense’s [14] semiquantitative calculation, approximately 40% of the entire fascia innervation consists of postganglionic sympathetic fibers. The majority of these fibers are vasoconstrictors because there are no targets in the fascia proper for the other sympathetic efferents (e.g., sudomotor, pilomotor, cardiomotor, secretomotor). Since sympathetic activity is higher when an individual is under psychologic stress, the dense sympathetic innervation may explain why many patients with low back pain report increased pain levels when they are under stress.

Finally, other fascial systems could be involved in pain generation. Indeed, Fede et al. [56] reported finding endoccannabinoid receptors 1 and 2 (CB1 and CB2) in various deep fasciae. It is well known that the endocannabinoid system plays an important role in pain modulation. In fact, the activation of CB1R is able to suppress pain signaling at the supraspinal, spinal and peripheral levels [57]. The endocannabinoid system can also modulate fibrosis and inflammation: its activation, in fact, can suppress proinflammatory cytokines such as IL-1beta and TNF-alpha and increase anti-inflammatory cytokines, providing antifibrotic activity [58]. Some researchers have attempted to employ pharmacotherapies (agonists/antagonists or cannabis used as a drug), to modify endocannabinoid levels in tissues (by inhibiting the enzymatic degradation of endocannabinoids), or to use manipulative treatments such as exercise or stretching that can activate or inactivate cannabinoid receptors to treat pain [59]. 

## 7. The Extracellular Matrix: The Fibrous Component

All the cells of the fascia are immersed in an abundant extracellular component, consisting of a three-dimensional, highly dynamic matrix, made up of collagens, proteoglycans/glycosaminoglycans, elastin, fibronectin, laminins, and several other glycoproteins. Together, these components form a complex network in which the cells reside, communicate and are involved in various cellular functions such as growth, differentiation and migration [60]. The two main classes of molecules found in the extracellular matrix are fibrous proteins and the aqueous component (ground substance), as explained above. Each component has different characteristics and functions. 

The *fibrous component* is fundamental for transmitting muscular force, connecting different segments and containing the structures [61]. It consists of collagen fibers (especially collagen types I and III), which provide a supporting framework of tissues and cells, and elastic fibers (elastin and fibrillin) which counterbalance the amount of collagen needed to allow tissues to cope with stretching and distension and permitting distribution of the stresses that maintain tissue resilience [62]. Moreover, the aponeurotic fasciae contain less than 1% of elastic fibers [50]. 

Different fasciae seem to present varied quantities of elastic fibers. The Van Gieson stain highlights a significant difference between the fascia lata and the deep fasciae of the upper limb. Indeed, many elastic fibers are present in the aponeurotic fasciae of the upper limb intermixed with the collagen fiber bundles, forming an irregular mesh. The elastic fibers are evident in the deep fascia of the inferior limb only in the loose connective tissue between the different fibrous layers and in the periphery of the fasciae [26]. According to Mense [14], the thoracolumbar fascia (TLF) is not elastic, and the only layer that contains few elastic fibers is the inner one. Since the layer is made up of loose connective tissue, elastic forces cannot build up in it. However, as described by Willard et al. [63], after deformation of the fascia, reversibility is possible because of the lattice grille-like arrangement of the collagen bundles in the middle layer. According to those investigators, the oblique collagen fiber bundles in the middle layer can slide against one other when the trunk is bent, and they can also slide back again. The single collagen fiber bundle is probably relatively stiff, but its spatial arrangement could provide a certain resilience. 

The type of collagen fibers present in the deep fasciae can change depending on various hormonal, mechanical and chemical inputs. Considered an effective therapeutic option for plantar fasciitis and myofascial disorders [64], extracorporeal shockwave treatment enhances fibroblast proliferation and differentiation by activating gene expression for transforming growth factor β1 and collagen types I and III [65]. Pavan et al. [66] demonstrated that aging increases the amount of type I collagen fibers in the extracellular matrix (ECM) of the epimysial fascia, and thus increase its stiffness. Slimani et al. [67] also demonstrated that immobilization causes pronounced muscle connective tissue thickening, affecting the elasticity/stiffness of the muscle. 

It has also been demonstrated that the fascial fibroblasts have specific receptors on the plasma membrane for estrogen and relaxin hormones, and that hormone levels in the blood can regulate the productions of collagen I, collagen III and fibrillin [68]. At a postmenopausal level of β-estradiol (~10 pg/mL), the fascial tissue became enriched in collagen-I (8.4% of positivity with a statistically significant variation from a starting percentage of 5.2 in control cells). Conversely, the amount of collagen III was lower with respect to that in the controls (1.5% of positivity with respect to 2.4% in the control cells) meaning that the fascial tissue was probably more rigid. However, when the hormone levels rose, e.g., during the ovulatory period or during pregnancy, the fascial tissue became more elastic and contained higher levels of collagen-III (6.8% or 6.7%, periovulatory and pregnancy concentrations, respectively) and fibrillin (3% and 3.6%, respectively, compared to 0.5% in the control) and there was a corresponding decrease in collagen I (1.9%). 

The presence of relaxin receptor 1 (RXFP1) and estrogen receptor-alpha (ERα) in the deep fascia also change throughout an individual’s lifetime and is lower in the postmenopausal period with respect to the premenopausal one [68]. These findings may explain some clinical differences detected in women of different age groups and why women tend to have different myofascial problems and myofascial pain after menopause [69]. When Vita et al. [70] analyzed the influence of hormonal changes on the deep fasciae during the menstrual cycle using ultrasound technology and shear wave elastography, they found statistically significant differences between the users and nonusers of hormonal contraceptives: the thoracolumbar fascia was thicker in the nonusers (*p* = 0.011), and the nonusers had higher maximal and mean stiffnesses of the fascia lata (*p* = 0.01 and 0.0095, respectively). Petrofsky and Lee [71] also demonstrated that plantar fascia elasticity modifications during the menstrual cycle can affect posture sway and tremor and lead to the potential risk of falling. 

## 8. The Extracellular Matrix: The Aqueous Matrix

The *water component* of the extracellular matrix is composed of a complex mixture of glycosaminoglycans (GAGs), most often covalently linked to proteins, forming proteoglycans and glycoproteins. Hyaluronan acid appears to be the most important GAG [72]. When Fede et al. [73] quantified the amount of HA in the various muscular fasciae, they found about 43 μg/g of HA in aponeurotic fasciae; there was a drastic decrease (about 6 μg/g) in epimysial fasciae, and an increase in the retinacula (90.4 μg/g). These variations correspond exactly with the various gliding functions of the fasciae, which change depending on the anatomical site: the aponeurotic fasciae, the same as the fascia lata of the thigh and the rectus sheath of the abdomen, are free to glide over the muscles. The epymisial fascia is a fibrous layer that is completely adherent to the underlying muscles and has fewer gliding properties [26]. The retinacula are specialized aponeurotic fasciae that surround the joints, where the highest levels of HA are found [51]. HA not only plays a fundamental role in regulating the properties of the fascial structures, but also in cell proliferation and mobility, inflammation and angiogenesis; it is also involved in various diseases such as cancer, diabetes, vascular alterations and others [74]. 

Many physical, mechanical, hormonal and pharmacological factors can influence the production of the various fibrous and GAG components of the fascial ECM. Recently Fede et al. [75] demonstrated that human fascial fibroblasts are able to produce HA-rich vesicles in vitro within a few hours of CB2 receptor agonist treatment. It was found that HA-rich vesicles were released into the extracellular environment leading to greater tissue fluidity. The study also showed that fascial cells respond to the endocannabinoid system by regulating and remodeling the formation of the ECM and, for the first time, demonstrated that beyond the central nervous system’s effect on pain perception, therapeutic applications of cannabinoids to treat myofascial pain syndromes may have a direct peripheral effect in remodeling the tissue and on its properties. Higher hyaluronan production after pharmacological stimulation constitutes only one example of how HA molecules can influence the properties of the fascial tissue. In general, HA influences all the biological activities of the ECM, as it has a fundamental lubricating function and facilitates smooth gliding between the densely packed collagen layers, between the deep fascia and muscle, and within the muscle itself [72,76]. It has a high turnover rate in human tissues due to the simultaneous action of synthetases (HAS1, HAS2, HAS3), hyaluronidases (HYAL) in their various isoforms and other degrading molecules, such as reactive oxygen species [77]. It can also bind and interact with a variety of receptors (CD44, RHAMM, LYVE-1) generating different signaling pathways [78]. 

HA polymers can range in size from a few kilodaltons to 8 MDa; its molecular size greatly influences the biological functions of the molecule [79]. In general, high molecular weight molecules have shown antiangiogenic, immunosuppressive, anti-inflammatory and tissue damage repair activities; smaller fragments are, on the contrary, proinflammatory and proangiogenic. HA can, however, alter density, concentration and viscosity depending on chemical or physical parameters—variations in temperature, the quantity of tissue water, the binding of proteins or the formation of crosslinks can change the chains of HA molecules causing consequent changes in viscosity and elasticity of HA-containing fluid. An increase of even two degrees centigrade due, for example, to a massage can cause a progressive break-up of the three-dimensional superstructure of HA chains, with a consequent decrease in viscosity [80]. The opposite can occur after intense physical exercise: muscle pH may reach a value of 6.60, with an increase of about 20% in HA viscosity [81]. As viscosity increases, so does the thickness of loose connective tissue due to high concentrations of sticky hyaluronan, but the gliding property of the fascial layer decreases, leading to less flexibility [24]. 

## 9. Perspectives 

Current research has shown that not all cells in the deep/muscular fasciae have the same properties. Functional characteristics of the cells are quite different, so the next step is to accurately identify the cell surface markers or transcription factors to distinguish the difference between them, which is particularly important to clarify the biological function. Future studies including larger numbers of samples will be able to quantify fasciacytes in the deep/muscular fasciae of different topographic districts and in various pathological conditions. Moreover, the quantification of HA, in the deep/muscular fasciae of different topographic districts and in various pathological conditions, will contribute to our knowledge of the pathophysiology of the deep/muscular fasciae. A better cellular and molecular knowledge may also be able to uncover changes which are invisible during clinical inspection and unforeseen by current clinical practice. Finally, being able to define the specific cellular and molecular structures involved in fascial dysfunctions would facilitate a more targeted approach to treatment and therapies.

## 10. Conclusions

The fascia is not only a band of connective tissue connecting the tissues of the human body, it is also a complex, vital structure that supports, covers, joins, penetrates, connects, interacts and responds to various kind of stimuli. The fascial system permeates the body, enabling all of its systems to operate in an integrated manner. The more we learn about it, the more we recognize its vitality and complexity. All the cells of the fascia and each component of the extracellular matrix play specific roles, can respond to various stimuli and can be modulated. Aging, exercise and hormonal factors can affect the fascial system. Future research will lead to more knowledge on the structure and behavior of the fasciae in normal healthy states as well as in pathological situations such as myofascial pain syndromes. A better understanding of its dynamics will in turn help us to identify rational treatments for pain syndromes associated with fascial tissues and guidelines for appropriate physical activity and healthy lifestyle.
